# 5,2′‐dibromo‐2,4′,5′‐trihydroxydiphenylmethanone attenuates LPS‐induced inflammation and ROS production in EA.hy926 cells via HMBOX1 induction

**DOI:** 10.1111/jcmm.13948

**Published:** 2018-10-24

**Authors:** Hong‐Xia Yuan, Xiu‐E Feng, En‐Li Liu, Rui Ge, Yuan‐Lin Zhang, Bao‐Guo Xiao, Qing‐Shan Li

**Affiliations:** ^1^ School of Public Health Science & Pharmaceutical Science Shanxi Medical University Taiyuan China; ^2^ Shanxi Key Laboratory of Innovative Drug for the Treatment of Serious Diseases Basing on the Chronic Inflammation, Shanxi University of Chinese medicine Taiyuan China

**Keywords:** 5,2′‐dibromo‐2,4′,5′‐trihydroxydiphenylmethanone, EA.hy926 cells, homeobox containing 1, lipopolysaccharide, reactive oxygen species

## Abstract

Inflammation and reactive oxygen species (ROS) are important factors in the pathogenesis of atherosclerosis (AS). 5,2′‐dibromo‐2,4′,5′‐trihydroxydiphenylmethanone (TDD), possess anti‐atherogenic properties; however, its underlying mechanism of action remains unclear. Therefore, we sought to understand the therapeutic molecular mechanism of TDD in inflammatory response and oxidative stress in EA.hy926 cells. Microarray analysis revealed that the expression of homeobox containing 1 (HMBOX1) was dramatically upregulated in TDD‐treated EA.hy926 cells. According to the gene ontology (GO) analysis of microarray data, TDD significantly influenced the response to lipopolysaccharide (LPS); it suppressed the LPS‐induced adhesion of monocytes to EA.hy926 cells. Simultaneously, TDD dose‐dependently inhibited the production or expression of IL‐6, IL‐1β, MCP‐1, TNF‐α, VCAM‐1, ICAM‐1 and E‐selectin as well as ROS in LPS‐stimulated EA.hy926 cells. HMBOX1 knockdown using RNA interference attenuated the anti‐inflammatory and anti‐oxidative effects of TDD. Furthermore, TDD inhibited LPS‐induced NF‐κB and MAPK activation in EA.hy926 cells, but this effect was abolished by HMBOX1 knockdown. Overall, these results demonstrate that TDD activates HMBOX1, which is an inducible protective mechanism that inhibits LPS‐induced inflammation and ROS production in EA.hy926 cells by the subsequent inhibition of redox‐sensitive NF‐κB and MAPK activation. Our study suggested that TDD may be a potential novel agent for treating endothelial cells dysfunction in AS.

## 1 INTRODUCTION

Reportedly, bromophenols (BPs), which are isolated from marine algae, exhibit interesting biological activities including anti‐oxidative,^1^, ^2^, ^3^ antibacterial,^4^, ^5^ anticancer,^6^ anti‐inflammatory,^7^ anti‐diabetic^8^ and glucose 6‐phosphate dehydrogenase inhibitory^9^ effects. Encouraged by these exciting pharmacological characteristics, our group synthesized a series of BP analogues, and several of our novel compounds exhibited high bioactivity both in vitro and in vivo.^10^, ^11^, ^12^, ^13^, ^14^, ^15^ Among these, TDD showed the most potent anti‐oxidant and cytoprotective properties,^14^ which may be related to its participation in the regulation of apoptosis and inflammation.^13^ In addition, pharmacological studies have demonstrated that TDD can ameliorate myocardial ischaemia‐reperfusion injury and prevent arteriosclerosis.^10^, ^11^ Taken together, these findings indicate that TDD could improve endothelial function, reduce oxidative stress and suppress inflammatory reactions. Thus, studies aiming towards understanding the molecular mechanisms underlying TDD activities are imperative.

HMBOX1, considered to be a transcription repressor, was first identified and synthesized from a human pancreatic cDNA library.^16^ HMBOX1 is expressed in many human tissues, including the cytoplasm of the human umbilical vein endothelial cells (HUVECs). The lack of HMBOX1 in HUVECs can induce cell apoptosis and inhibit cell autophagy, which are indispensable for HUVEC survival.^17^ In addition, HMBOX1 is essential for the maintenance of endothelial function and has the potential to be a novel therapeutic target for atherosclerosis (AS).^18^ However, the precise role of HMBOX1 in the context of anti‐inflammatory and anti‐oxidative effects of TDD in EA.hy926 cells remains unknown.

Endothelial dysfunction is a critical factor in the pathogenesis of AS.^19^, ^20^, ^21^ An increasing number of studies have demonstrated the close association of vascular inflammatory response and oxidative stress with endothelial dysfunction.^22^, ^23^, ^24^ Thus, the inhibition of vascular inflammatory response and oxidative stress may be a promising therapeutic approach in the treatment of AS and other cardiovascular diseases. To investigate the key molecules and pathways of TDD, gene expression profiles of control and TDD‐treated EA.hy926 cells were studied using microarray analysis. Our results indicated that HMBOX1 was dramatically upregulated in TDD‐treated EA.hy926 cells. One of the biological processes most significantly influenced by TDD was the response to lipopolysaccharide (LPS); this result is in agreement with the results of previous investigations.^13^ Reportedly, LPS can initiate vascular inflammatory response and oxidative stress.^25^, ^26^ Therefore, we sought to understand the correlation between HMBOX1 activation and the anti‐inflammatory and antioxidant effects of TDD in LPS‐treated EA.hy926 cells and further explored the possible underlying mechanisms.

## 2 MATERIALS AND METHODS

### 2.1 Materials

TDD was synthesized in our laboratory as previously reported (Supplementary Material online, Figure S1, 99% purity).^14^ LPS was purchased from Sigma‐Aldrich (St. Louis, MO, USA). Antibodies for phosphor (p)‐ERK1/2, ERK1/2, p‐p38, p38, p‐JNK, JNK, ICAM‐1, VCAM‐1, E‐selectin and NF‐κB/p65 were purchased from Cell Signaling Technology (Beverly, MA, USA). TRIzol reagent, a PrimeScript™ RT reagent kit and an SYBR Premix Ex Taq™ II kit were obtained from TaKaRa Bio, Inc. (Shiga, Japan). Lamin B Rabbit Monoclonal Antibody, a nuclear protein extraction kit, and an NF‐κB Activation‐Nuclear Translocation Assay Kit were obtained from Biotechnology Co. (Shanghai, China). The antibody for HMBOX1 was obtained from Proteintech Group Inc. (Wuhan, China). ELISA kits for IL‐6 and TNF‐α, β‐actin antibody, horseradish peroxidase‐conjugated secondary antibodies and enhanced chemiluminescence (ECL) detection kit were obtained from Boster Biotech Co., Ltd. (Wuhan, China).

### 2.2 Cell cultivation

Cells of the human‐derived EAhy.926 EC line and THP‐1 monocytes were obtained from the Cell Bank of the Chinese Academy of Sciences (Shanghai, China). The cells were cultured in high‐glucose DMEM or RPMI 1640 medium according to the manufacturer's recommendations.

### 2.3 Cell viability and apoptosis

Cell viability was determined by MTT quantitative colorimetric assay as previously reported.^27^ EA.hy926 cells were treated with TDD at various concentrations (0‐80 μmol/L) for 24 hours in 96‐well plates. Cells were subsequently incubated with 10 μL MTT at 37°C for 4 hours. After the medium was removed, the cell precipitates were dissolved in 100 μL of DMSO and analysed at 570 nm on a Bio‐Rad Model 680 Microplate Reader (Hercules, CA, USA). Simultaneously, cell apoptosis was analysed using an Annexin V‐FITC Apoptosis Kit (Life Technologies, Eugene, OR, USA).^27^ Briefly, cells were treated with various concentrations of TDD for 24 hours in a six‐well dish. The cells were digested, washed with PBS and resuspended in binding buffer. Then, the cells were incubated with Annexin V‐FITC and PI in the dark for 15 minutes before analysis using a flow cytometer (Becton Dickinson, Franklin Lakes, NJ, USA).

### 2.4 Microarray analysis

Total RNA was extracted from EA.hy926 cells, which were either untreated or exposed 10 μmol/L TDD for 1 hour. An Agilent 2100 bioanalyzer (Agilent, Santa Clara, CA, USA) was used to evaluate RNA purity and concentration. The EA.hy926 cell gene expression profiles of different groups were analysed using the Agilent Human 8 × 60 K.

### 2.5 Cell adhesion assay

Monocytes adhering to EA.hy926 cells were evaluated by using THP‐1 cells as previously described.^28^ Briefly, EA.hy926 cells were treated with TDD (0, 5, 10 and 20 μmol/L) for 4 hours before the addition of LPS (1 μg/mL) for 12 hours in 96‐well plates. Calcein AM‐labelled THP‐1 cells were then added to EA.hy926 cells for 1 hour and non‐adherent THP‐1 cells were removed by washing with PBS. The fluorescence of bound monocytes was measured using a multi‐detection microplate reader (Variskan Flash; Thermo Scientific, Waltham, MA, USA) at 490 and 515 nm.

### 2.6 Cytokine assays

The concentrations of the cytokines IL‐6 and TNF‐α in the supernatants were measured using ELISA kits according to the manufacturer's recommendations.

### 2.7 Measurement of ROS generation

The production of intracellular reactive oxygen species (ROS) was evaluated using the DCFH‐DA probe (Sigma‐Aldrich) as previously described.^27^ Then, DCF fluorescence was detected by flow cytometric analysis at 488 and 525 nm using a BD Biosciences flow cytometer.

### 2.8 Quantitative real‐time polymerase chain reaction

Total cell RNA was extracted using TRIzol reagent. The cDNA was amplified from 1.0 μg total RNA using the PrimeScript RT™ reagent kit. Primer sequences used in this study are shown in Table 1. Quantitative analysis of mRNA expression was performed on a StepOnePlus™ PCR System (Applied Biosystems, Foster City, CA, USA) using the SYBR Premix Ex Taq™ II Kit. Raw data were calculated and normalized to the mRNA expression levels of GAPDH.

**Table 1:** List of primer sequences

Primer name	Primer sequence
TNF‐α (forward)	5′‐CCTGTGAGGAGGACGAACAT‐3′
TNF‐α (reverse)	5′‐TTTGAGCCAGAAGAGGTTGAG‐3′
MCP‐1 (forward)	5′‐TCAGCCAGATGCAATCAATG‐3′
MCP‐1 (reverse)	5′‐AGATCTCCTTGGCCACAATG‐3′
IL‐1β (forward)	5′‐TGGCAGAAAGGGAACAGAAA‐3′
IL‐1β (reverse)	5′‐CTGGCTGATGGACAGGAGAT‐3′
IL‐6 (forward)	5′‐GTGTGAAAGCAGCAAAGAG‐3′
IL‐6 (reverse)	5′‐CTCCAAAAGACCAGTGATG‐3′
ICAM‐1 (forward)	5′‐TCACCTATGGCAACGACTCC‐3′
ICAM‐1 (reverse)	5′‐GTGTCTCCTGGCTCTGGTTC‐3′
VCAM‐1 (forward)	5′‐GAAGGTGGCTCTGTGACCAT‐3′
VCAM‐1 (reverse)	5′‐AAAGGTGCTGTAGATTCCCATT‐3′
E‐Selection (forward)	5′‐CTGAGTCCTGCTCCTTCCAA‐3′
E‐Selection (reverse)	5′‐CTTCGGTGTAGCCCATTTGT‐3′
HMBOX1 (forward)	5′‐AGCATGGGTCAGAGGTCATACAG‐3′
HMBOX1 (reverse)	5′‐GGAAAGTACCAGATGTGGCAG‐3′
GAPDH (forward)	5′‐GCACCGTCAAGGCTGAGAAC‐3′
GAPDH (reverse)	5′‐TGGTGAAGACGCCAGTGGA‐3′

### 2.9 Western blot analysis

Extracts from cell cytoplasm and nuclear proteins were prepared as previously described.^29^ Sample proteins (30 μg) were separated by SDS‐PAGE and transferred to nitrocellulose membranes. After the membranes were blocked with 5% non‐fat milk, they were probed overnight at 4°C with the indicated antibodies. Then, membranes were washed and incubated with horseradish peroxidase‐conjugated secondary antibodies as previously described.^27^, ^29^ Immunoreactivity was detected by ECL reagents and data were analysed using Adobe Photoshop CC software.

### 2.10 Confocal microscopy

EA.hy926 cells were cultured in four‐chambered coverglass and treated with TDD (20 μmol/L) for 4 hours before the addition of LPS (1 μg/mL) for a further 2 hours. An NF‐κB nuclear translocation assay was performed using an NF‐κB activation‐nuclear translocation assay kit. Briefly, after the cells were fixed and blocked, they were incubated with NF‐κB p65 antibody at 4°C overnight. Next, the cells were incubated with a Cy3‐conjugated secondary antibody at room temperature for 1 hour and stained with DAPI for 5 minutes. Immunofluorescence was observed using an Olympus Fluoview FV1000 confocal microscope (Waltham, MA, USA).

### 2.11 HMBOX1 siRNA transfection

The HMBOX1 gene silencer was designed and synthesized by GenePharma (Shanghai, China). Transfection experiments were performed using 50 nmol/L siRNA and siRNA‐mate Reagent (GenePharma) according to the manufacturer's protocol.

### 2.12 Statistical analysis

All experiments were conducted in triplicates. All results are expressed as the mean ± SD. The data were analysed using the Student's *t*‐test and differences were considered statistically significant at *P *<* *0.05.

## 3 RESULTS

### 3.1 TDD had no cytotoxicity at optimal concentrations

In this study, the cytotoxicity of TDD in the EA.hy926 cells was investigated with the MTT assay. The results showed that TDD had no cellular toxicity at concentrations of up to 20 μmol/L over 24 hours (Supplementary material online, Figure S2). Consistent with the MTT assay, TDD did not induce cell apoptosis at concentrations of 5‐20 μmol/L compared with EA.hy926 cells treated without TDD (Supplementary material online, Figure S3). Therefore, in the subsequent experiments, TDD was used at a concentration between 5 and 20 μmol/L.

### 3.2 Microarray analysis of TDD‐treated EA.hy926 cells

**Figure 1:**
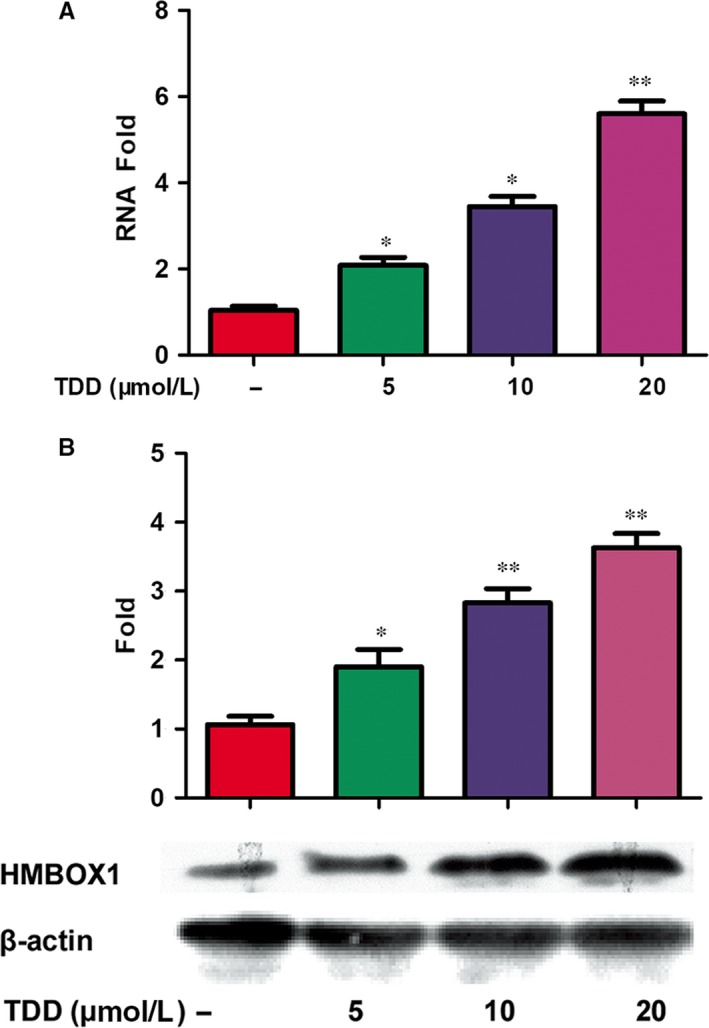
TDD‐mediated regulation of HMBOX1 expression in EA.hy926 cells. (A) HMBOX1 mRNA expression was analyzed by FQ‐PCR and normalized mRNA values relative to GAPDH levels were plotted. (B) Effect of TDD on HMBOX1 protein levels was evaluated by western blot with analysis of β‐actin expression as loading control. The data show the mean ± S.D. of three independent experiments. *P < 0.05 and **P < 0.01, compared to the TDD (‐) group.

**Figure 2:**
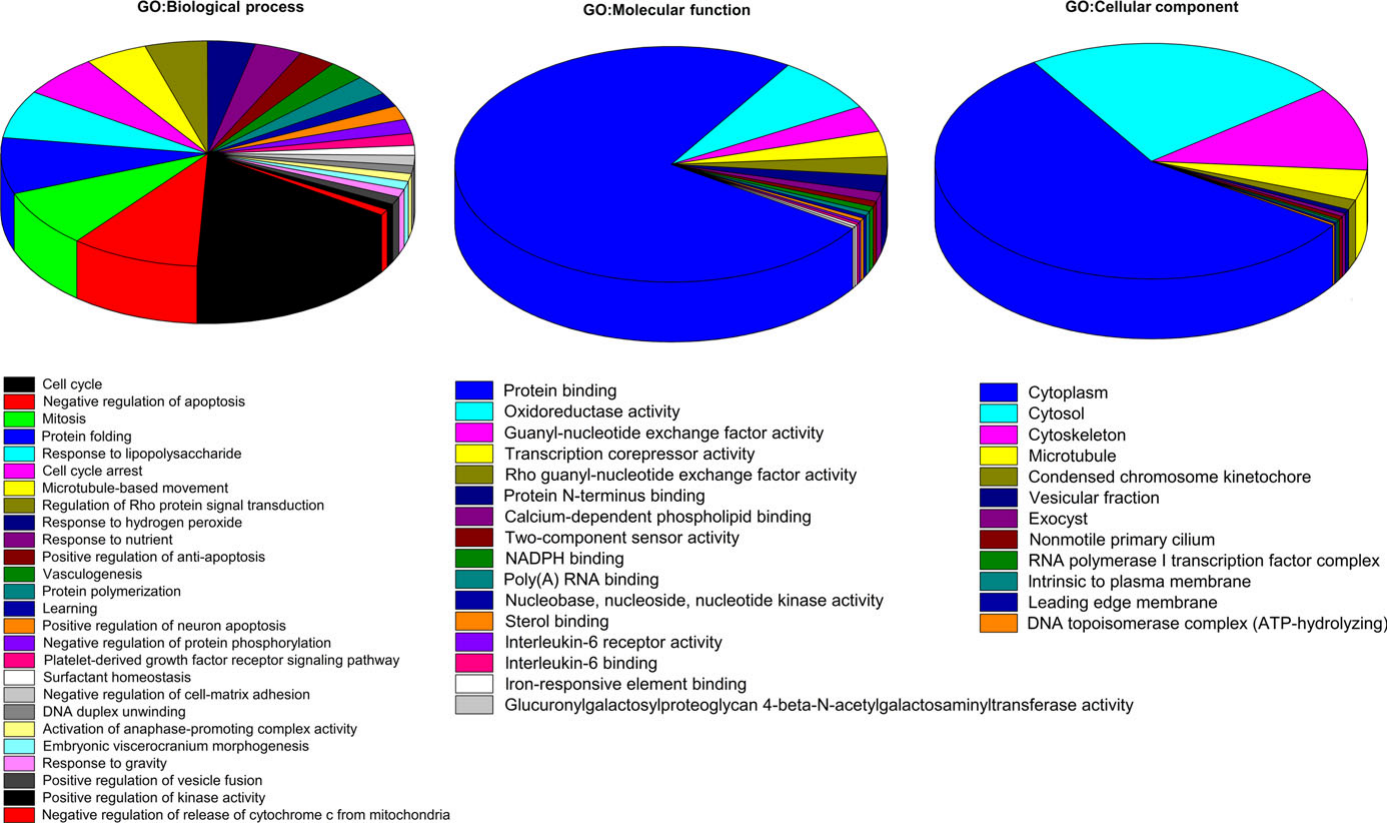
The differentially expressed genes in TDD‐treated EA.hy926 cells were analyzed according to gene ontology in three dimensions: (A) biological processes, (B) molecular functions and (C) cellular localization.

To investigate the target‐related genes and pathways in TDD‐treated EA.hy926 cells, gene expression profiles of control and TDD‐treated EA.hy926 cells were studied by microarray analysis. Approximately 42 545 mRNAs with differential changes induced by TDD were identified, with a two‐fold or more increase in the expression of 2964 mRNAs. The expressions of some representative genes were evaluated by fluorescence quantitative polymerase chain reaction (Supplementary material online, Figure S4). Among them the most prominently regulated gene induced by TDD was HMBOX1. Therefore, we focused study on HMBOX1. As shown in Figure 1, treatment with TDD in EA.hy926 cells significantly increased the expression level of HMBOX1 mRNA and protein in a dose‐dependent manner. Biological processes related to genes with differential expression were done using GO analysis. Among them the response to the LPS pathway was shown to be significantly impacted by TDD treatment (Figure 2).

### 3.3 TDD inhibited LPS‐induced THP‐1 adhesion

**Figure 3:**
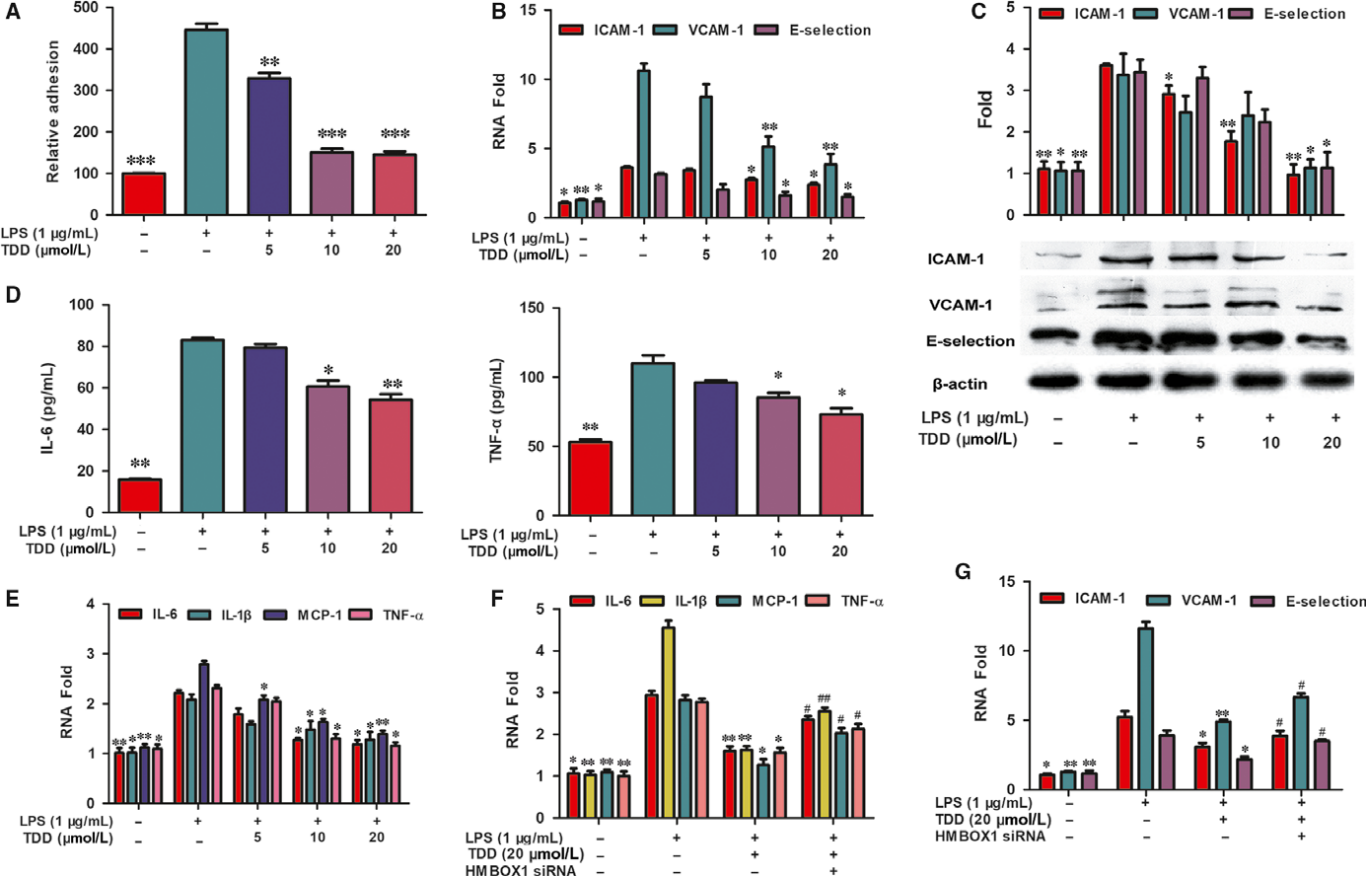
Effects of TDD on LPS‐stimulated monocyte adhesion, adhesion molecule and pro‐inflammatory cytokines expression. (A) THP‐1 cells were labeled with the fluorescent probe and the adhesion was determined. (B) The gene expression of VCAM‐1, ICAM‐1 and E‐selection levels were determined by real‐time PCR and normalized to GAPDH. (C) The expression of VCAM‐1, ICAM‐1 and E‐selection were measured by Western blot assay. The β‐actin protein level was considered as an internal control. (D) The production of TNF‐α and IL‐6 were measured by ELISA analysis. (E) The mRNA expression of IL‐6, IL‐1β, MCP‐1 and TNF‐α were determined by FQ‐PCR. (F, G) Effects of TDD on LPS‐stimulated the IL‐6, IL‐1β, MCP‐1, TNF‐α, ICAM‐1, VCAM‐1 and E‐selection mRNA expression were determined by FQ‐PCR with or without transfecting HMBOX1 siRNA. The data show the mean ± SD of three independent experiments. *P <* *0.05, **P <* *0.01 and ***P <* *0.001, compared to the LPS (+) group. ^#^ *P *<* *0.05 and ^##^ *P *<* *0.01, compared to the LPS (+) TDD (+) group

Inflammation‐induced mononuclear cell adhesion to the endothelium is believed to be one of the earliest events in the development of AS.^30^ To assess the effect of TDD on this monocyte‐endothelium adhesion, experiments were conducted using fluorescence‐labelled THP‐1 cells. Exposing EA.hy926 cells to LPS significantly increased the adhesion of the THP‐1 cells to EA.hy926 cells (Figure 3A). TDD pre‐treatment considerably prevented the LPS‐induced adhesion of THP‐1 cells to EA.hy926 cells, and the treatment with 20 μmol/L TDD reduced the LPS‐induced THP‐1 adhesion by more 50%.

### 3.4 TDD inhibited LPS‐induced cell adhesion molecule expression and pro‐inflammatory cytokine production via HMBOX1 activation

The increased expression of cell adhesion molecule (CAMs), such as VCAM‐1, ICAM‐1 and E‐selectin, may contribute to the recruitment of inflammatory monocytes into the vascular wall and the initiation of AS.^31^ Therefore, the effect of TDD on the expression levels of CAM mRNAs and proteins in LPS‐stimulated EA.hy926 cells was examined. As shown in Figure 3B,C, LPS exposure significantly increased the expression levels of CAMs, and TDD inhibited this effect in a dose‐dependent manner.

Further, the effects of TDD on the production of pro‐inflammatory cytokines such as IL‐6 and TNF‐α, was analysed using ELISA. The results indicated that secretion of IL‐6 and TNF‐α increased in LPS‐stimulated EA.hy926 cells and that TDD slightly inhibited this secretion (Figure 3D). In addition, whether TDD could reduce the levels of pro‐inflammatory cytokine mRNAs in LPS‐treated EA.hy926 cells was examined. As shown in Figure 3E, transcription levels of IL‐6, IL‐1β, MCP‐1 and TNF‐α decreased following TDD treatment compared with those following LPS treatment.

To determine whether TDD could inhibit LPS‐induced inflammation via HMBOX1 signalling, HMBOX1 knockdown analysis was performed using siRNA technology. The efficacy of siRNA interference was assessed by RT‐PCR and western blotting, and the most effective target was selected (Supplementary material online, Figure S5A‐C). Our results demonstrated that the effects of TDD on the expression of inflammatory mediators in LPS‐treated‐EA.hy926 cells were abolished by HMBOX1 siRNA treatment (Figure 3F,G), indicating that TDD prevents LPS‐induced inflammation through HMBOX1 activation.

### 3.5 TDD suppressed LPS‐induced ROS production via HMBOX1 activation in EA.hy926 cells

**Figure 4:**
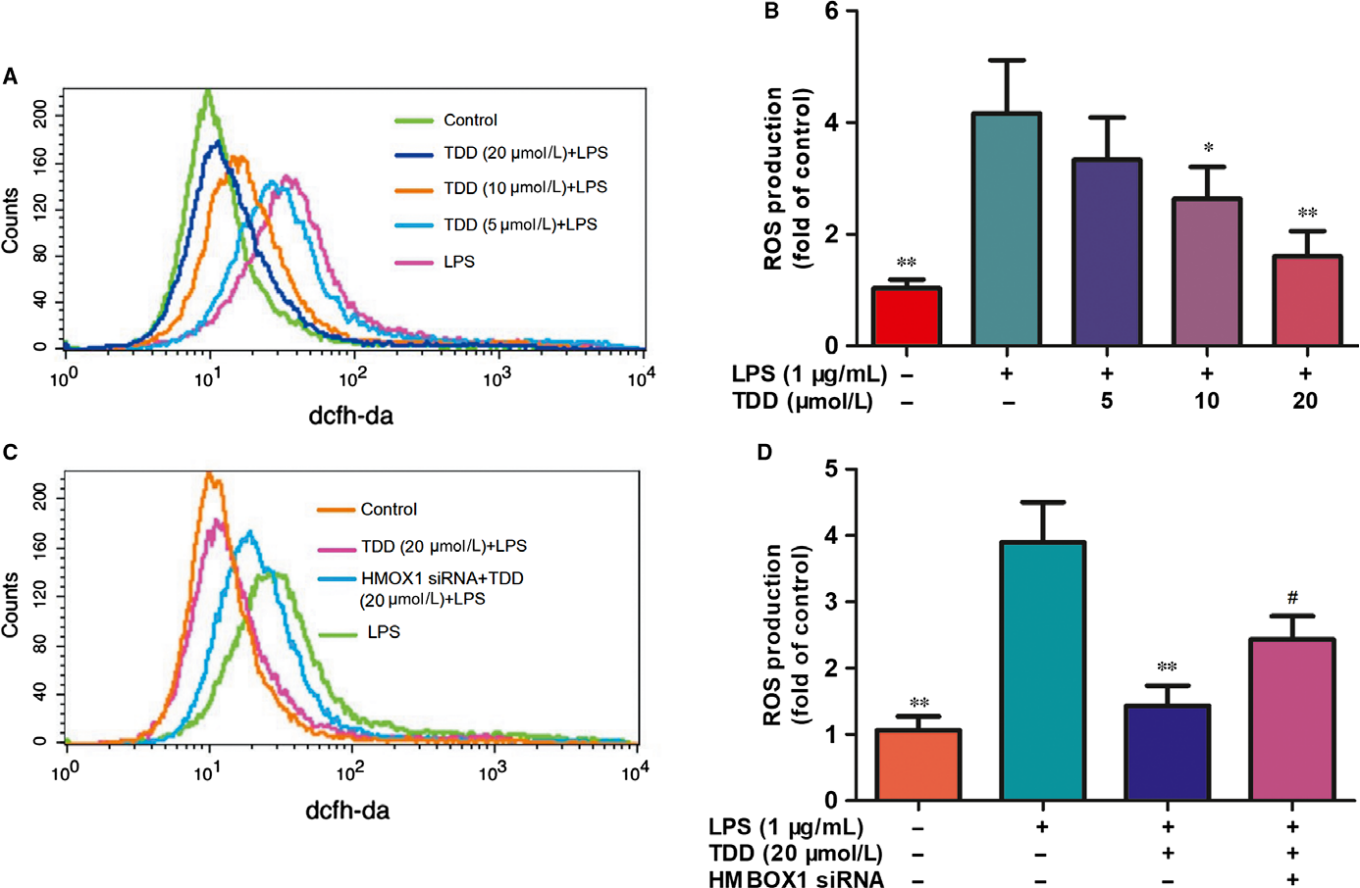
Effects of TDD on LPS‐induced ROS expression in EA.hy926 Cells. The ROS production was detected using the DCFH‐DA probe; (A, B) EA.hy926 Cells were treated with TDD for 4 h before addition of LPS (1 μg/mL) for another 30 min. The DCF fluorescence intensity was detected by flow cytometric analysis. (C, D) Effects of TDD on LPS‐induced ROS expression in EA.hy926 Cells with or without transfecting HMBOX1 siRNA. The data show the mean ± SD of three independent experiments. *P <* *0.05, **P <* *0.01 and ***P <* *0.001, compared to the LPS (+) group. ^#^ *P *<* *0.05, compared to the LPS (+) TDD (+) group

To assess the anti‐oxidant properties of TDD, ROS levels were measured. LPS significantly increased ROS, but TDD treatment (20 μmol/L) effectively suppressed ROS production (Figure 4A,B). In addition, the effects of TDD on LPS‐induced ROS expression were abolished by HMBOX1 siRNA treatment (Figure 4C,D), indicating that TDD prevents LPS‐induced ROS production through HMBOX1 activation.

### 3.6 TDD inhibited LPS‐induced NF‐κB activation by activating HMBOX1

**Figure 5:**
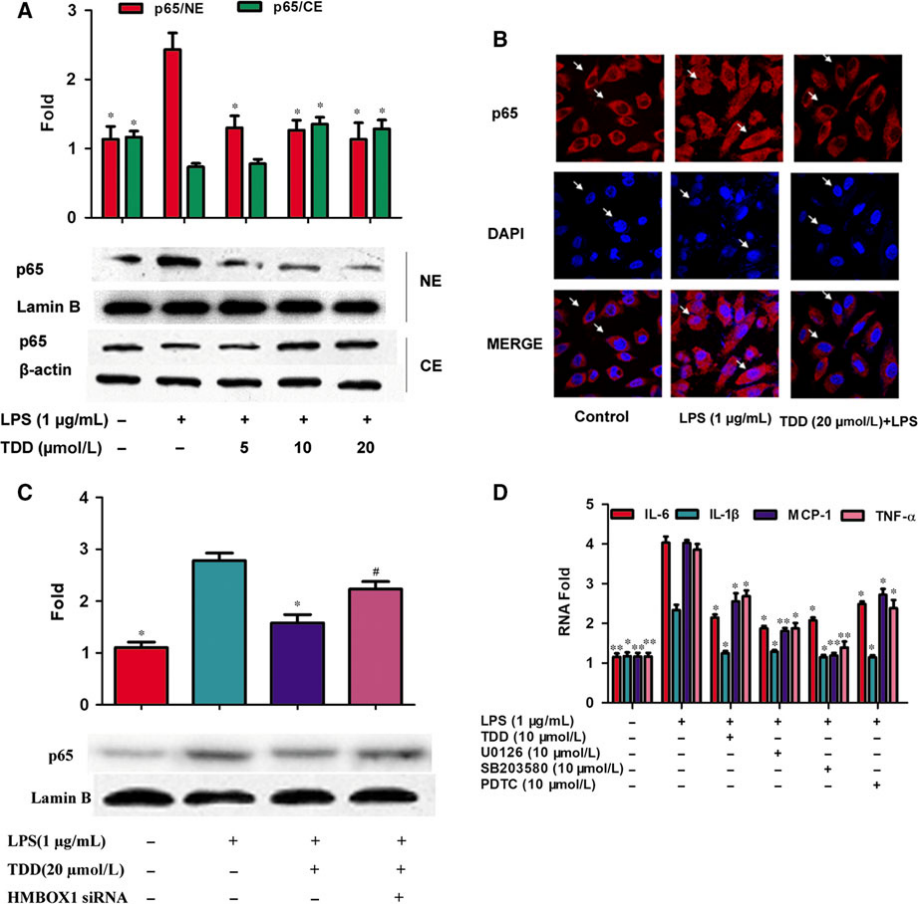
Effects of TDD on nuclear translocation of NF‐κB/p65 via HMBOX1 activation in LPS‐stimulated EA.hy926 Cells. NE, nuclear extracts; CE, cytoplasmic extracts. (A) The cells were treated with TDD for 4 h before addition of LPS (1 μg/mL) for another 2 h. Cytoplasmic and nuclear levels of NF‐κB p65 were detected by Western blotting to analyze the translocation of NF‐κB. Lamin B and β‐actin were used as loading controls for nuclear and cytosolic protein fractions, respectively. (B) Immunofluorescent imaging shows a TDD‐mediated suppression of LPS‐mediated nuclear translocation of NF‐κB p65 in EA.hy926 Cells. The arrow indicates the position of NF‐Κb p65 (magnification, ×1000). (C) Effects of TDD on nuclear translocation of NF‐κB/p65 in LPS‐stimulated EA.hy926 Cells with or without transfecting HMBOX1 siRNA. (D) Effects of NF‐κB, ERK1/2 and p38 MAPK inhibitors on the transcription of IL‐6, IL‐1β, MCP‐1 and TNF‐α in LPS‐stimulated EA.hy926 cells. The data show the mean ± SD of three independent experiments. *P <* *0.05 and **P <* *0.01, compared to the LPS (+) group. ^#^ *P *<* *0.05 compared to the LPS (+) TDD (+) group

Previous studies have shown that NF‐κB is a key regulator of pro‐inflammatory cytokines.^32^ Therefore, the effect of TDD on LPS‐induced NF‐κB activation in EA.hy926 cells was examined. Our results indicate that TDD pre‐treatment inhibited LPS‐increased p65 NF‐κB translocation to the nuclear fraction in EA.hy926 cells (Figure 5A). The inhibitory effect of TDD on LPS‐induced NF‐kB p65 nuclear translocation was consistent with protein expression levels and further confirmed using confocal microscopy (Figure 5B).

Next, HMBOX1 knockdown analysis was used to investigate whether HMBOX1 influenced the effect of TDD on LPS‐induced NF‐κB activation. As shown in Figure 5C, HMBOX1 silencing reversed the repressive effects of TDD on LPS‐induced NF‐κB activation. These results indicate that TDD could suppress the LPS‐induced activation of NF‐κB at least in part by activating HMBOX1. Furthermore, PDTC (an inhibitor of NF‐κB) suppressed the transcription levels of IL‐6, IL‐1β, MCP‐1 and TNF‐α in LPS‐stimulated EA.hy926 cells (Figure 5D).

### 3.7 TDD suppressed LPS‐induced phosphorylation of MAPK via HMBOX1 activation

**Figure 6:**
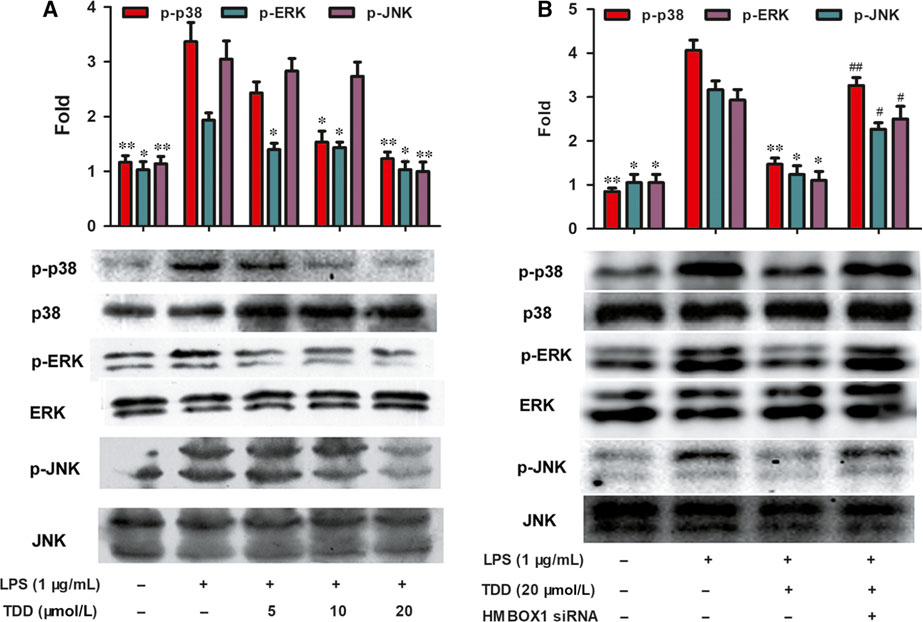
Effects of TDD on LPS‐stimulated phosphorylation of MAPKs via HMBOX1 activation in EA.hy926 Cells. (A) EA.hy926 Cells were treated with TDD for 4 h before addition of LPS (1 μg/mL) for another 30 min. Relative protein expressions were analyzed by Western blot analysis. (B) Effects of TDD on MAPKs activation in LPS‐stimulated EA.hy926 cells with or without transfecting HMBOX1 siRNA. The data show the mean ± SD of three independent experiments. *P <* *0.05 and **P <* *0.01, compared to the LPS (+) group. ^#^ *P *<* *0.05 and ^##^ *P *<* *0.01, compared to the LPS (+) TDD (+) group

Because MAPKs play important roles in regulating inflammation, whether TDD could inhibited the phosphorylation of MAPKs in LPS‐treated EA.hy926 cells was investigated. As shown in Figure 6A, phosphorylation of p38, ERK1/2 and JNK was significantly increased in LPS‐treated cells, but TDD pre‐treatment (5‐20 μmol/L) inhibited this effect. To investigate whether HMBOX1 is involved in TDD suppression of MAPK phosphorylation, HMBOX1 was knocked down using siRNA. As shown in Figure 6B, HMBOX1 silencing reversed the repressive effects of TDD on MAPK phosphorylation in LPS‐treated EA.hy926 cells.

Taken together, our results indicate that TDD attenuates LPS‐induced MAPK phosphorylation via HMBOX1 activation. Moreover, U0126 (an inhibitor of ERK1/2) and SB203580 (an inhibitor of p38 MAPK) inhibited the transcription of IL‐6, IL‐1β, MCP‐1 and TNF‐α in LPS‐stimulated EA.hy926 cells (Figure 5D).

## 4 DISCUSSION

Inflammation and oxidative stress play key roles in the progression of cardiovascular diseases, including AS.^33^, ^34^, ^35^ Therefore, the inhibition of inflammatory response and ROS production may be beneficial in preventing the development of AS.^33^ Endothelial cell dysfunction is another major cause AS development.^19^, ^20^, ^21^ Although primary HUVECs are considered to be the best model for endothelial function studies, they may undergo phenotypic change with passages and may require specialized culture media for growth. Nowadays, the EA.hy926 cell line is widely used to study the protective effects of various chemical substances on the vascular endothelium in vitro,^36^ owing to the ability to preserve the biological characteristics and cell function of primary HUVECs such as endothelin converting enzyme,^37^ VIII‐related antigen (VIIIR:Ag),^38^, ^39^ prostacyclin^40^ and so on.

Atherosclerosis is usually a chronic inflammatory disease of the vessel walls, and its systemic inflammation may be mimicked by exposure to LPS and *Porphyromonas gingivalis* endotoxins.^41^ Systemic inflammation mediated by the infusion of endotoxins results in the formation and development of atheromatous lesions in animals.^42^, ^43^, ^44^ Epidemiological studies also indicate that an elevated serum LPS level constitutes a critical risk factor for the development of AS in humans.^45^, ^46^, ^47^ A pivotal mechanism of AS aggravation is proposed to be a part of an endothelial injury pathway induced by LPS.^48^, ^49^

Pharmacological studies have demonstrated that TDD can ameliorate myocardial ischaemia‐reperfusion injury and prevent arteriosclerosis,^10^, ^11^ indicating that TDD could improve endothelial function, suppress the inflammatory reaction and reduce oxidative stress. To investigate TDD target genes, the microarray analysis of differences between TDD‐treated and control EA.hy926 cells was conducted. In this study, approximately 42 545 mRNAs with differential changes induced by TDD were identified in EA.hy926 cells. Among these, the highest fold change in upregulation induced by TDD was in HMBOX1.

On the basis of the results of the GO analysis of microarray data, biological processes influenced by TDD were determined to be involved in the response to LPS, which is in agreement with previous results.^13^ Exposing vascular endothelial cells to LPS generated inflammatory cytokines and ROS.^25^, ^26^, ^50^ Here, we investigated the correlation between HMBOX1 activation and the anti‐inflammatory and antioxidant effects of TDD in LPS‐treated EA.hy926 cells and further explored the possible underlying mechanisms.

Because TDD can prevent arteriosclerosis, we hypothesized that TDD might suppress LPS‐triggered interactions between monocytes and vascular cells, which is an early step in the development of AS.^30^ CAMs are the key factors involved in the regulation of enhanced endothelium‐monocyte interactions leading to inflammation.^31^, ^51^, ^52^ Meanwhile, impaired endothelial cells can release inflammatory cytokines, such as MCP‐1, IL‐6, IL‐1β and TNF‐α, which can trigger stronger inflammatory vascular responses, accelerating the development of AS.^53^, ^54^ In the present study, TDD suppressed LPS‐induced increase in VCAM‐1, ICAM‐1 and E‐selectin mRNA levels and protein expression. In addition, TDD also significantly downregulated the production or expression of IL‐6, IL‐1β, MCP‐1 and TNF‐α in LPS‐stimulated EA.hy926 cells in a concentration‐dependent manner. Other studies have reported that ROS also contributed to endothelial adhesion molecule expression and cytokine production.^25^, ^55^ To further validate whether the effect of TDD on the inhibition of inflammation and ROS production in LPS‐stimulated EA.hy926 cells was mediated through HMBOX1, HMBOX1 silencing was conducted. Our results indicated for the first time that anti‐inflammatory and anti‐oxidative effects of TDD were abolished when HMBOX1 was silenced.

NF‐κB is a known redox‐sensitive transcription factor and can be activated by ROS,^56^, ^57^ which has been shown to regulate the expression of CAMs and the production of inflammatory cytokines, which are involved in the progression of AS.^58^, ^59^ When cells are stimulated by LPS, activated IKK phosphorylates IκBα and the phosphorylated IκBα is subsequently degraded through ubiquitination. The liberated NF‐κB then enters the nucleus, where it can bind to specific DNA motifs and trigger an inflammatory response.^56^ Thus, the effect of TDD on NF‐κB activation in LPS‐stimulated EA.hy926 cells was detected by western blotting. Results showed that TDD treatment inhibited NF‐κB activation. To further confirm these results, an immunofluorescence assay revealed that TDD treatment inhibited the translocation of NF‐B p65 to the nucleus in LPS‐stimulated EA.hy926 cells.

In addition to NF‐kB, this secretion of CAMs and pro‐inflammatory cytokines is always dependent on the activation of MAPKs.^58^, ^60^ Our results indicated that TDD treatment inhibits the LPS‐stimulated activation of p38 and ERK1/2. In addition, when LPS‐stimulated EA.hy926 cells were exposed to PDTC (a selective NF‐κB inhibitor), SB203580 (a selective p38 MAPK inhibitor) or U0126 (a selective MEK1/2 inhibitor), the levels of IL‐6, IL‐1β, MCP‐1 and TNF‐α mRNA decreased. Therefore, our results confirmed that TDD inhibited the release of inflammatory factors by inhibiting the LPS‐induced activation of NF‐κB and MAPKs. Moreover, this inhibitory effect was attenuated by HMBOX1 siRNA, suggesting that TDD prevented LPS‐induced NF‐κB and MAPK activation via HMBOX1 signal activation.

**Figure 7:**
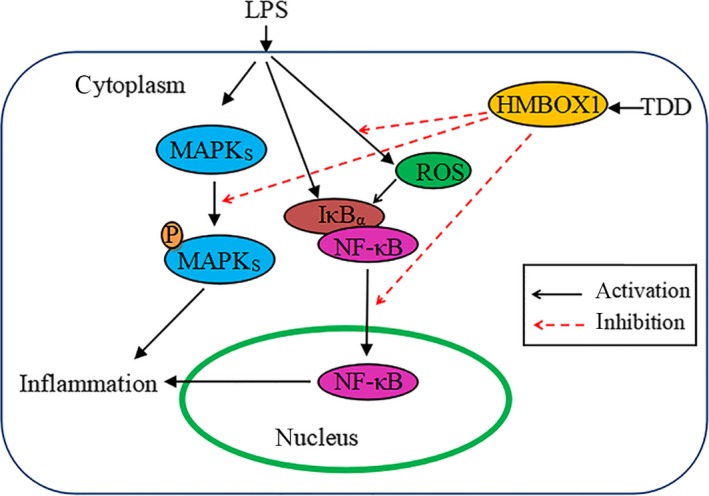
Diagram of the possible mechanisms of TDD‐induced effects in LPS‐stimulated EA.hy926 Cells. TDD as a HMBOX1 activator to suppress LPS‐stimulated inflammation and ROS production, subsequent inhibition of redox‐sensitive NF‐κB and MAPK activation

In conclusion, our results demonstrate that TDD inhibits the LPS‐mediated adhesion of monocytes to EA.hy926 cells and suppresses the levels of CAMs and pro‐inflammatory cytokines as well as ROS production in LPS‐stimulated EA.hy926 cells. Moreover, the protective, anti‐inflammatory and anti‐oxidative effects of TDD are likely mediated via the activation of HMBOX1, which contributes to the inhibition of redox‐sensitive NF‐κB and MAPK pathways (Figure 7). In conclusion, TDD may be a potential novel anti‐inflammatory and anti‐oxidative drug for treating endothelial cell dysfunction in AS.

## AUTHOR CONTRIBUTIONS

Qing‐Shan Li conceived and designed the research. Hong‐Xia Yuan wrote the main text and prepared all of the Figures. Bao‐Guo Xiao helped draft the final manuscript. Rui Ge and En‐li Liu helped prepare experiments. Xiu‐e Feng, Yuan‐Lin Zhang and Hong‐Xia Yuan performed the experiments. Hong‐Xia Yuan and Xiu‐e Feng contributed equally as first authors.

## CONFLICT OF INTEREST

All authors declare no competing interests.

## References

[1] Olsen EK , Hansen E , Isaksson J , Andersen JH . Cellular antioxidant effect of four bromophenols from the red algae, *Vertebrata lanosa* . Mar Drugs. 2013;11:2769‐2784. 23921722 10.3390/md11082769 PMC3766864

[2] Xu X , Yin L , Gao L , et al. Two new bromophenols with radical scavenging activity from marine red alga *Symphyocladia latiuscula* . Mar Drugs. 2013;11:842‐847. 23528951 10.3390/md11030842 PMC3705374

[3] Li K , Li XM , Gloer JB , Wang BG . New nitrogen‐containing bromophenols from the marine red alga *Rhodomela confervoides* and their radical scavenging activity. Food Chem. 2012;135:868‐872. 22953798 10.1016/j.foodchem.2012.05.117

[4] Popplewell WL , Northcote PT . Colensolide A: a new nitrogenous bromophenol from the New Zealand marine red alga *Osmundaria colensoi* . Tetrahedron Lett. 2009;50:6814‐6817.

[5] Xu N , Fan X , Yan X , et al. Antibacterial bromophenols from the marine red alga *Rhodomela confervoides* . Phytochemistry. 2003;62:1221‐1224. 12648540 10.1016/s0031-9422(03)00004-9

[6] Liu M , Zhang W , Wei J , et al. Marine bromophenol bis(2,3‐dibromo‐4,5‐dihydroxybenzyl) ether, induces mitochondrial apoptosis in K562 cells and inhibits topoisomerase I in vitro. Toxicol Lett. 2012;211:126‐134. 22484147 10.1016/j.toxlet.2012.03.771

[7] Wiemer DF , Idler DD , Fenical W . Vidalols A and B, new anti‐inflammatory bromophenols from the Caribbean marine red alga *Vidalia obtusaloba* . Experientia. 1991;47:851‐853. 1915767 10.1007/BF01922471

[8] Liu X , Li X , Gao L , et al. Extraction and PTP1B inhibitory activity of bromophenols from the marine red alga *Symphyocladia latiuscula* . Chin J Oceanol Limnol. 2011;29:686‐690.

[9] Mikami D , Kurihara H , Kim SM , Takahashi K . Red algal bromophenols as glucose 6‐phosphate dehydrogenase inhibitors. Mar Drugs. 2013;11:4050‐4057. 24152564 10.3390/md11104050 PMC3826149

[10] Li QS , Wang Y , Feng XE , Han LG . The Application of Two Bromophenols and their Medicinal Salts as the Drugs Against the Myocardial Ischemia Reperfusion Injury. Beijing, China: State Intellectual Property Office of the P.R.C; 2013.

[11] Li QS , Feng XE , Ban SR . The Application of Polyhydroxyl Bromo‐ Diphenylketone Compounds and their Derivatives in the Treatment and Prevention of Atherosclerosis. Beijing, China: State Intellectual Property Office of the P.R.C; 2014.

[12] Feng XE , Zhao WY , Ban SR , et al. Structure‐activity relationship of halophenols as a new class of protein tyrosine kinase inhibitors. Int J Mol Sci. 2011;12:6104‐6115. 22016647 10.3390/ijms12096104 PMC3189771

[13] Li J , Feng X , Ge R , et al. Protective effect of 2,4′,5′‐trihydroxyl‐5,2′‐dibromo diphenylmethanone, a new halophenol, against hydrogen peroxide‐induced EA.hy926 cells injury. Molecules. 2015;20:14254‐14264. 26251890 10.3390/molecules200814254 PMC6332007

[14] Zhao W , Feng X , Ban S , et al. Synthesis and biological activity of halophenols as potent antioxidant and cytoprotective agents. Bioorg Med Chem Lett. 2010;20:4132‐4134. 20621727 10.1016/j.bmcl.2010.05.068

[15] Zheng F , Ban S , Feng X , et al. Synthesis and biological activities of new halophenols. Med Chem. 2013;9:303‐311. 22946534 10.2174/1573406411309020013

[16] Chen S , Saiyin H , Zeng X , et al. Isolation and functional analysis of human HMBOX1, a homeobox containing protein with transcriptional repressor activity. Cytogenet Genome Res. 2006;114:131‐136. 16825764 10.1159/000093328

[17] Ma H , Su L , Yue H , et al. HMBOX1 interacts with MT2A to regulate autophagy and apoptosis in vascular endothelial cells. Sci Rep. 2015;5:15121. 26456220 10.1038/srep15121 PMC4600982

[18] Ma H , Su L , Zhang S , et al. Inhibition of ANXA7 GTPase activity by a small molecule promotes HMBOX1 translation of vascular endothelial cells in vitro and in vivo. Int J Biochem Cell Biol. 2016;79:33‐40. 27506770 10.1016/j.biocel.2016.08.010

[19] Buttari B , Profumo E , Businaro R , et al. Oxidized haemoglobin‐driven endothelial dysfunction and immune cell activation: novel therapeutic targets for atherosclerosis. Curr Med Chem. 2013;20:4806‐4814. 23834168 10.2174/09298673113209990162

[20] Gimbrone MA Jr , Garcia‐Cardena G . Endothelial cell dysfunction and the pathobiology of atherosclerosis. Circ Res. 2016;118:620‐636. 26892962 10.1161/CIRCRESAHA.115.306301 PMC4762052

[21] Lu H , Daugherty A . Atherosclerosis. Arterioscler Thromb Vasc Biol. 2015;35:485‐491. 25717174 10.1161/ATVBAHA.115.305380 PMC4511379

[22] Dinh QN , Drummond GR , Sobey CG , Chrissobolis S . Roles of inflammation, oxidative stress, and vascular dysfunction in hypertension. Biomed Res Int. 2014;2014:406960. 25136585 10.1155/2014/406960 PMC4124649

[23] Sprague AH , Khalil RA . Inflammatory cytokines in vascular dysfunction and vascular disease. Biochem Pharmacol. 2009;78:539‐552. 19413999 10.1016/j.bcp.2009.04.029 PMC2730638

[24] Loppnow H , Buerke M , Werdan K , Rose‐John S . Contribution of vascular cell‐derived cytokines to innate and inflammatory pathways in atherogenesis. J Cell Mol Med. 2011;15:484‐500. 21199323 10.1111/j.1582-4934.2010.01245.x PMC3922371

[25] Zhang M , Pan H , Xu Y , et al. Allicin decreases lipopolysaccharide‐induced oxidative stress and inflammation in human umbilical vein endothelial cells through suppression of mitochondrial dysfunction and activation of Nrf2. Cell Physiol Biochem. 2017;41:2255‐2267. 28456799 10.1159/000475640

[26] Li C , Ma D , Chen M , et al. Ulinastatin attenuates LPS‐induced human endothelial cells oxidative damage through suppressing JNK/c‐Jun signaling pathway. Biochem Biophys Res Commun. 2016;474:572‐578. 27109479 10.1016/j.bbrc.2016.04.104

[27] Feng XE , Liang TG , Gao J , et al. Heme oxygenase‐1, a key enzyme for the cytoprotective actions of halophenols by upregulating Nrf2 expression via activating Erk1/2 and PI3K/Akt in EA.hy926 cells. Oxid Med Cell Longev. 2017;2017:1‐13. 10.1155/2017/7028478 PMC5488237 28694915

[28] Huang CS , Lin AH , Yang TC , et al. Shikonin inhibits oxidized LDL‐induced monocyte adhesion by suppressing NFkappaB activation via up‐regulation of PI3K/Akt/Nrf2‐dependent antioxidation in EA.hy926 endothelial cells. Biochem Pharmacol. 2015;93:352‐361. 25541286 10.1016/j.bcp.2014.12.005

[29] Yang CM , Huang SM , Liu CL , Hu ML . Apo‐8’‐lycopenal induces expression of HO‐1 and NQO‐1 via the ERK/p38‐Nrf2‐ARE pathway in human HepG2 cells. J Agric Food Chem. 2012;60:1576‐1585. 22260728 10.1021/jf204451n

[30] Bozic M , Alvarez A , de Pablo C , et al. Impaired vitamin D signaling in endothelial cell leads to an enhanced leukocyte‐endothelium interplay: implications for atherosclerosis development. PLoS ONE. 2015;10:e0136863. 26322890 10.1371/journal.pone.0136863 PMC4556440

[31] Bhat OM , Uday Kumar P , Harishankar N , et al. Interleukin‐18‐induced cell adhesion molecule expression is associated with feedback regulation by PPAR‐gamma and NF‐kappaB in Apo E‐/‐ mice. Mol Cell Biochem. 2017;428:119‐128. 28176248 10.1007/s11010-016-2922-8

[32] Chen X , Xiu M , Xing J , et al. Lanthanum chloride inhibits LPS mediated expressions of pro‐inflammatory cytokines and adhesion molecules in HUVECs: involvement of NF‐kappaB‐Jmjd3 signaling. Cell Physiol Biochem. 2017;42:1713‐1724. 28743103 10.1159/000479439

[33] Garcia N , Zazueta C , Aguilera‐Aguirre L . Oxidative stress and inflammation in cardiovascular disease. Oxid Med Cell Longev. 2017;2017:5853238. 28536646 10.1155/2017/5853238 PMC5426074

[34] Wang L , Huang Z , Huang W , et al. Inhibition of epidermal growth factor receptor attenuates atherosclerosis via decreasing inflammation and oxidative stress. Sci Rep. 2017;8:45917. 28374780 10.1038/srep45917 PMC5379239

[35] Siti HN , Kamisah Y , Kamsiah J . The role of oxidative stress, antioxidants and vascular inflammation in cardiovascular disease (a review). Vascul Pharmacol. 2015;71:40‐56. 25869516 10.1016/j.vph.2015.03.005

[36] Li S , Ning H , Ye Y , et al. Increasing extracellular Ca(2 + ) sensitizes TNF‐alpha‐induced vascular cell adhesion molecule‐1 (VCAM‐1) via a TRPC1/ERK1/2/NFkappaB‐dependent pathway in human vascular endothelial cells. Biochim Biophys Acta. 2017;1864:1566‐1577. 10.1016/j.bbamcr.2017.06.001 28583863

[37] Ahn K , Pan S , Beningo K , Hupe D . A permanent human cell line (EA.hy926) preserves the characteristics of endothelin converting enzyme from primary human umbilical vein endothelial cells. Life Sci. 1995;56:2331‐2341. 7791520 10.1016/0024-3205(95)00227-w

[38] Bouïs D , Hospers GAP , Meijer C , et al. Endothelium in vitro: a review of human vascular endothelial cell lines for blood vessel‐related research. Angiogenesis. 2001;4:91‐102. 11806248 10.1023/a:1012259529167

[39] Edgell CJ , McDonald CC , Graham JB . Permanent cell line expressing human factor VIII‐related antigen established by hybridization. Proc Natl Acad Sci USA. 1983;80:3734‐3737. 6407019 10.1073/pnas.80.12.3734 PMC394125

[40] Suggs JE , Madden MC , Friedman M , Edgell CJ . Prostacyclin expression by a continuous human cell line derived from vascular endothelium. Blood. 1986;68:825‐829. 3092887

[41] Stoll LL , Denning GM , Weintraub NL . Potential role of endotoxin as a proinflammatory mediator of atherosclerosis. Arterioscler Thromb Vasc Biol. 2004;24:2227‐2236. 15472123 10.1161/01.ATV.0000147534.69062.dc

[42] Reifenberg K , Lehr HA , Fan J , et al. Endotoxin accelerates atherosclerosis independent of complement activation. Thromb Res. 2009;123:653‐658. 18692867 10.1016/j.thromres.2008.06.017

[43] Gitlin JM , Loftin CD . Cyclooxygenase‐2 inhibition increases lipopolysaccharide‐induced atherosclerosis in mice. Cardiovasc Res. 2009;81:400‐407. 18948273 10.1093/cvr/cvn286 PMC2639107

[44] Engelmann MG , Redl CV , Nikol S . Recurrent perivascular inflammation induced by lipopolysaccharide (endotoxin) results in the formation of atheromatous lesions in vivo. Lab Invest. 2004;84:425‐432. 14968125 10.1038/labinvest.3700065

[45] Wiedermann CJ , Kiechl S , Dunzendorfer S , et al. Association of endotoxemia with carotid atherosclerosis and cardiovascular disease. J Am Coll Cardiol. 1999;34:1975‐1981. 10588212 10.1016/s0735-1097(99)00448-9

[46] Losito A , Kalidas K , Santoni S , et al. Association of the ‐159C/T polymorphism of the endotoxin receptor (CD14) with carotid artery disease and cardiovascular mortality in dialysis patients. Blood Purif. 2005;23:128‐133. 15640605 10.1159/000083207

[47] Videm V , Svennevig JL , Fosse E , et al. Plasma endotoxin concentrations during cardiac surgery may be related to atherosclerosis. Perfusion. 2000;15:421‐426. 11001164 10.1177/026765910001500503

[48] Liao W . Endotoxin: possible roles in initiation and development of atherosclerosis. J Lab Clin Med. 1996;128:452‐460. 8900288 10.1016/s0022-2143(96)90042-6

[49] Pesonen E , Kaprio E , Rapola J , et al. Endothelial cell damage in piglet coronary artery after intravenous administration of *E. coli* endotoxin. A scanning and transmission electron‐microscopic study. Atherosclerosis. 1981;40:65‐73. 7025844 10.1016/0021-9150(81)90124-6

[50] Fu Y , Hu X , Cao Y , et al. Saikosaponin a inhibits lipopolysaccharide‐oxidative stress and inflammation in human umbilical vein endothelial cells via preventing TLR4 translocation into lipid rafts. Free Radic Biol Med. 2015;89:777‐785. 26475038 10.1016/j.freeradbiomed.2015.10.407

[51] Ao M , Wu L , Zhou X , Chen Y . Methyl‐beta‐cyclodextrin impairs the monocyte‐adhering ability of endothelial cells by down‐regulating adhesion molecules and caveolae and reorganizing the actin cytoskeleton. Biol Pharm Bull. 2016;39:1029‐1034. 27251506 10.1248/bpb.b16-00047

[52] Giunzioni I , Bonomo A , Bishop E , et al. Cigarette smoke condensate affects monocyte interaction with endothelium. Atherosclerosis. 2014;234:383‐390. 24747113 10.1016/j.atherosclerosis.2014.03.029

[53] Fatkhullina AR , Peshkova IO , Koltsova EK . The role of cytokines in the development of atherosclerosis. Biochemistry (Mosc). 2016;81:1358‐1370. 27914461 10.1134/S0006297916110134 PMC5471837

[54] Kollgaard T , Enevold C , Bendtzen K , et al. Cholesterol crystals enhance TLR2‐ and TLR4‐mediated pro‐inflammatory cytokine responses of monocytes to the proatherogenic oral bacterium *Porphyromonas gingivalis* . PLoS ONE. 2017;12:e0172773. 28235036 10.1371/journal.pone.0172773 PMC5325525

[55] Kim IS , Yang EJ , Shin DH , et al. Effect of arazyme on the lipopolysaccharideinduced inflammatory response in human endothelial cells. Mol Med Rep. 2014;10:1025‐1029. 24842222 10.3892/mmr.2014.2231

[56] Lingappan K . NF‐kappaB in oxidative stress. Curr Opin Toxicol. 2018;7:81‐86. 29862377 10.1016/j.cotox.2017.11.002 PMC5978768

[57] Gloire G , Legrand‐Poels S , Piette J . NF‐kappaB activation by reactive oxygen species: fifteen years later. Biochem Pharmacol. 2006;72:1493‐1505. 16723122 10.1016/j.bcp.2006.04.011

[58] Huang M , Zeng S , Zou Y , et al. The suppression of bromodomain and extra‐terminal domain inhibits vascular inflammation by blocking NF‐kappaB and MAPK activation. Br J Pharmacol. 2017;174:101‐115. 27774624 10.1111/bph.13657 PMC5341496

[59] Jung JY , Woo SM , Kim WJ , et al. Simvastatin inhibits the expression of inflammatory cytokines and cell adhesion molecules induced by LPS in human dental pulp cells. Int Endod J. 2017;50:377‐386. 27003335 10.1111/iej.12635

[60] Bi C , Jiang Y , Fu T , et al. Naringin inhibits lipopolysaccharide‐induced damage in human umbilical vein endothelial cells via attenuation of inflammation, apoptosis and MAPK pathways. Cytotechnology. 2016;68:1473‐1487. 27006302 10.1007/s10616-015-9908-3 PMC4960195

