# Supplementary material for: 5,2′‐dibromo‐2,4′,5′‐trihydroxydiphenylmethanone attenuates LPS‐induced inflammation and ROS production in EA.hy926 cells via HMBOX1 induction
Source: J Cell Mol Med. 2018 Oct 24;23(1):453–63. doi: 10.1111/jcmm.13948 (PMC6307801; doi:10.1111/jcmm.13948)
Supplement: Supplementary file 1 [file JCMM-23-453-s001.doc]

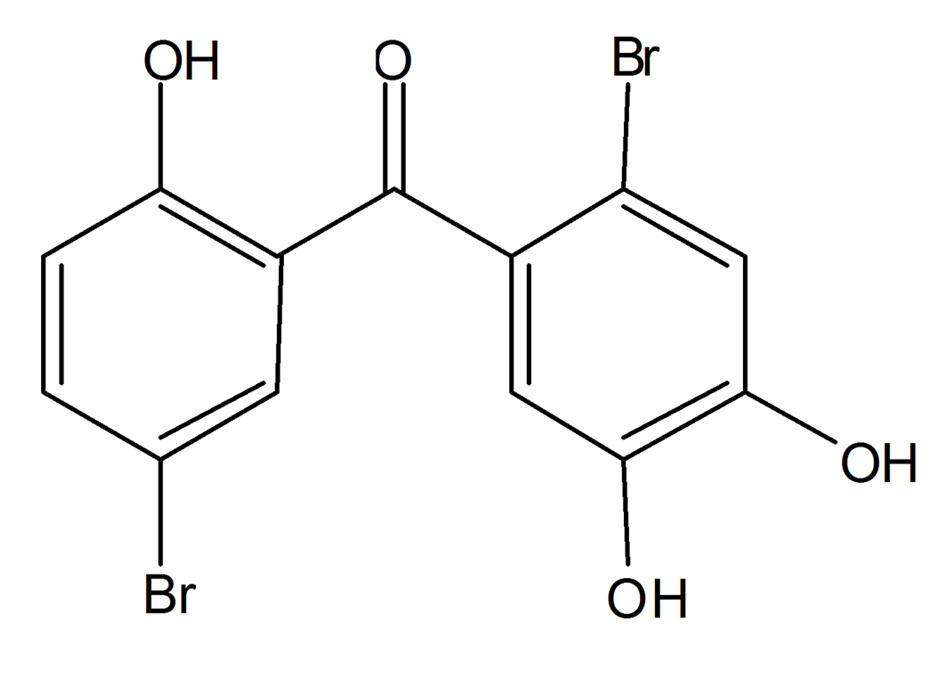


Fig. S1. Chemical structures of TDD.


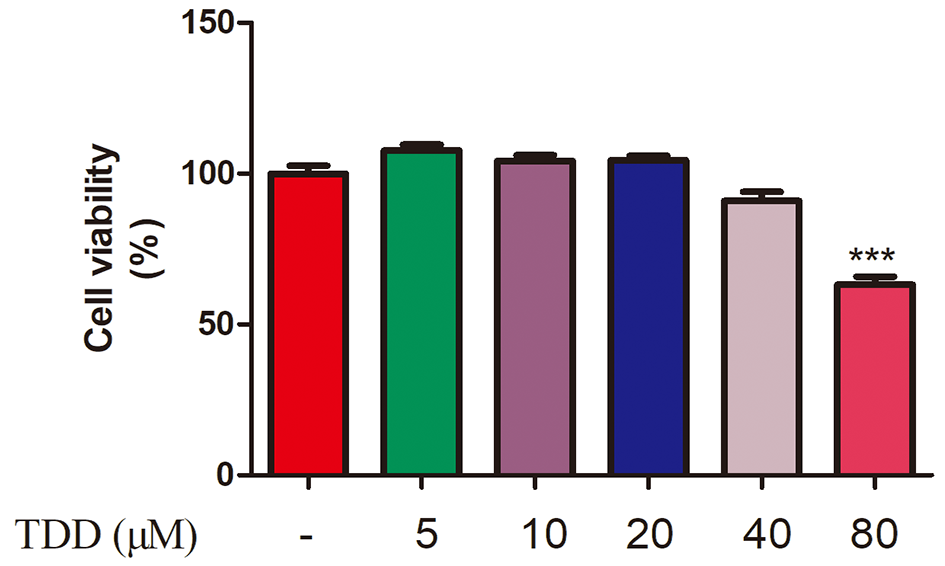


Fig. S2. Effect of TDD on the cell viability of EA.hy926 Cells. EA.hy926 Cells were treated with TDD at various concentrations for 24 h. Cell viability was determined by MTT assay as described in the Materials and methods section. The data show the mean ± S.D. of three independent experiments. ^***^*P*<0.001, compared to the TDD (-) group.


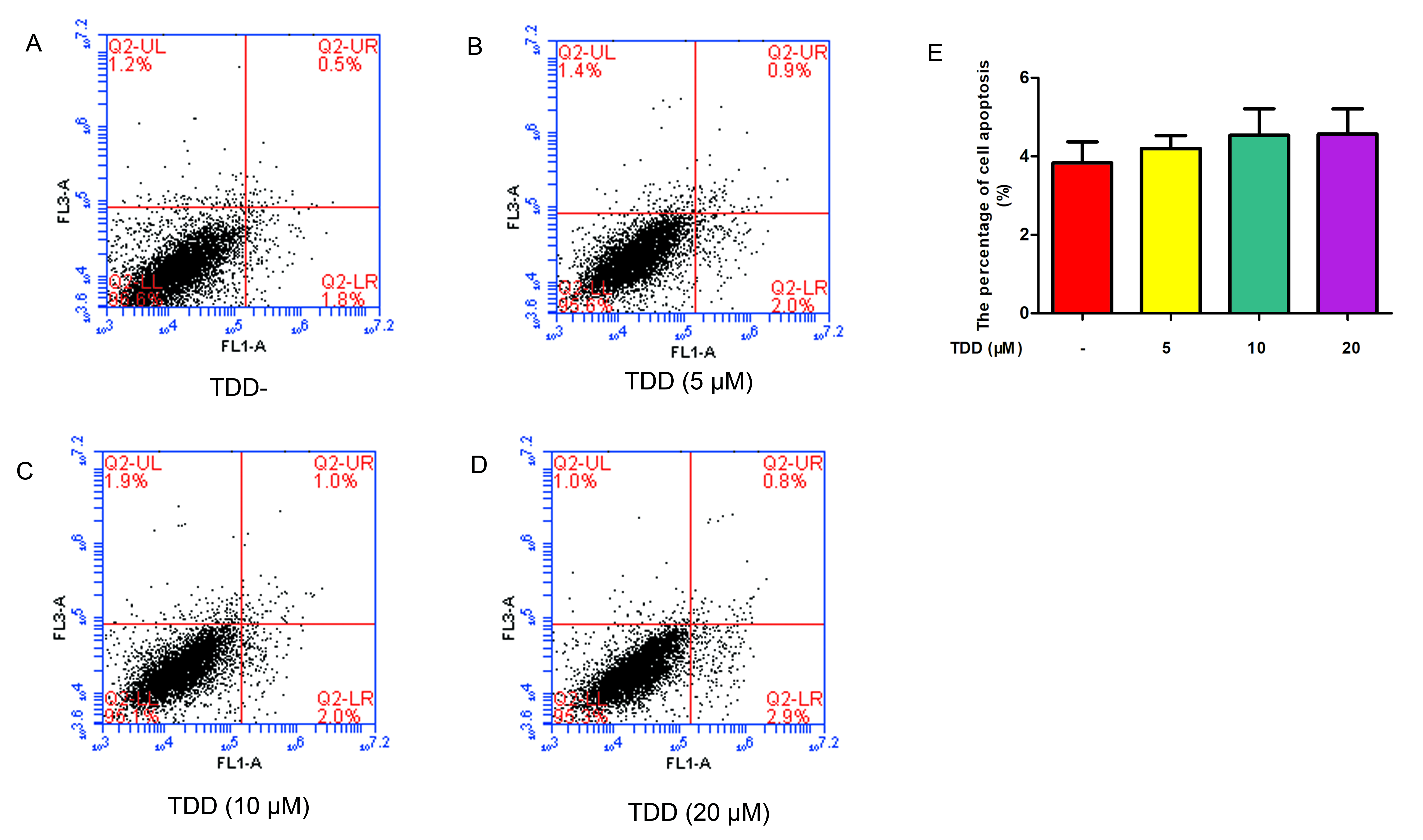


Fig. S3. The percentage of apoptotic EA.hy926 cells induced by TDD. EA.hy926 cells were treated with TDD at various concentrations for 24 h. Fig.3A–3D: Cell apoptosis was monitored by FACS using an Annexin V-FITC Apoptosis Kit. Fig. 3E: Results were expressed as a percentage of apoptosis cells and were the mean ± SEM from three independent experiments performed in triplicate.


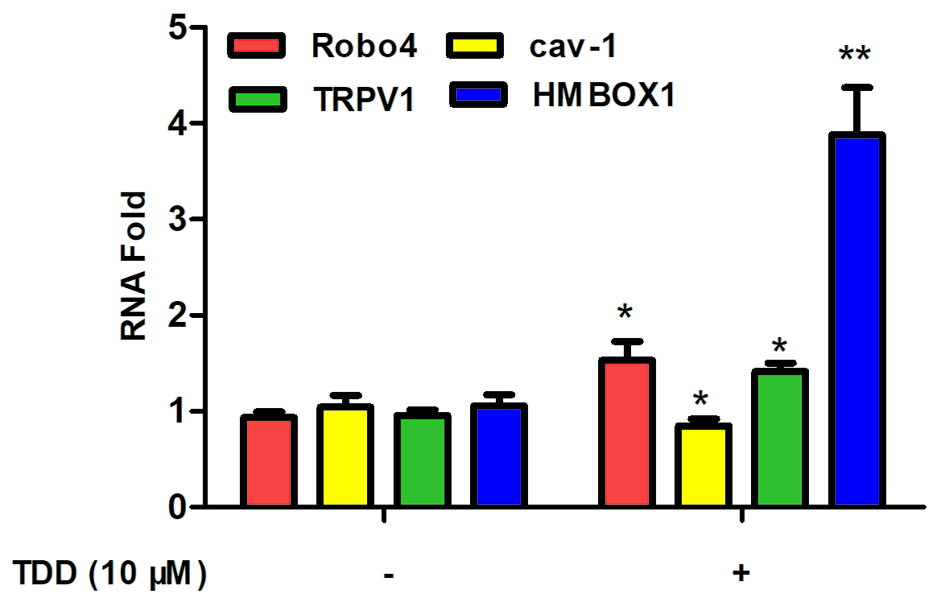


Fig. S4. RT-PCR analysis of the genes Robo4, cav-1, TRPV1 and HMBOX1 genes influenced by TDD in EA.hy926 cells. Data are shown as mean ± SEM from three independent experiments. **P*<0.05 and ***P*<0.01, compared to the TDD (-) group.


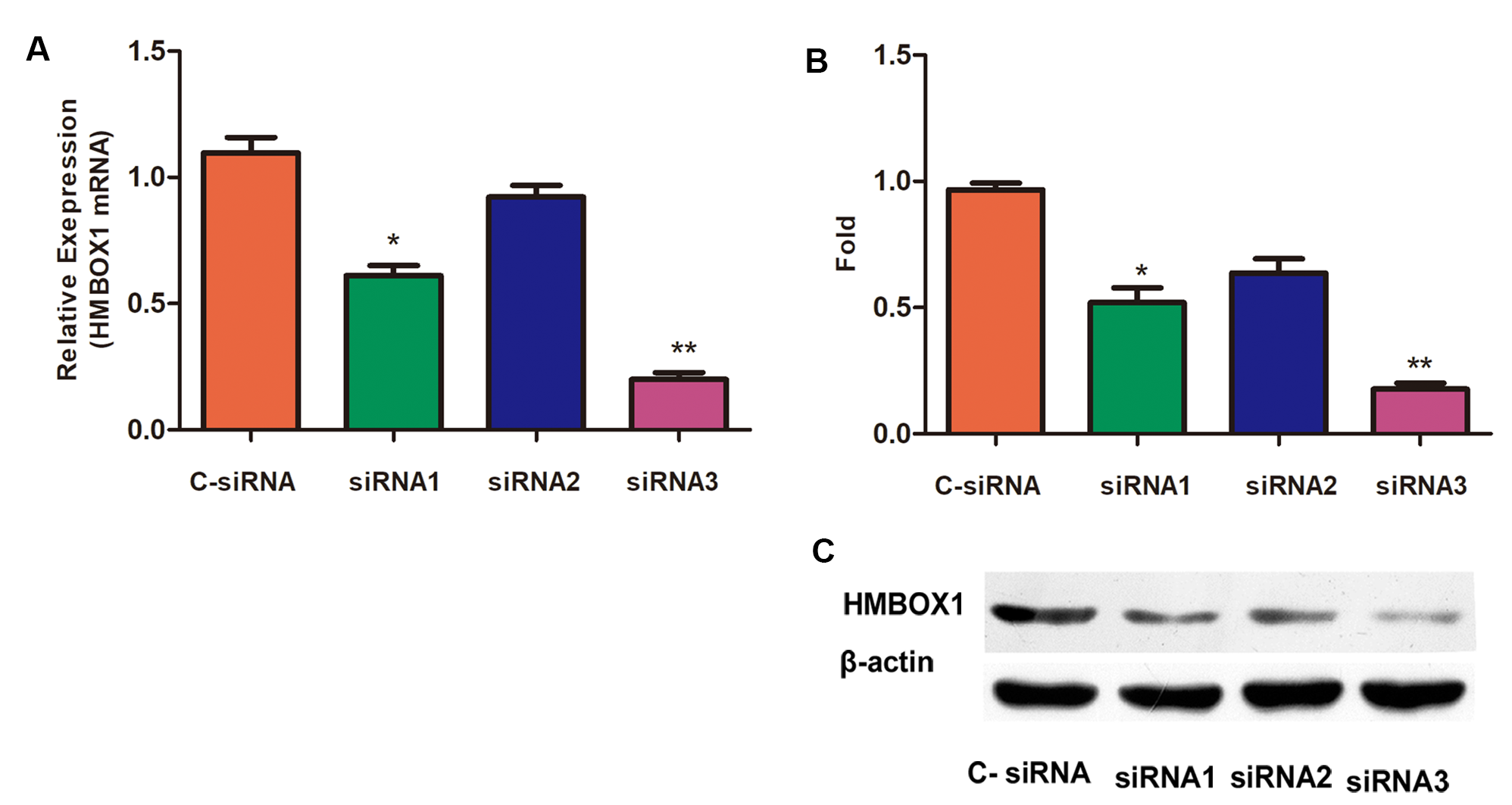


Fig. S5. The efficacy of siRNA interference was assessed using real-time PCR and western blotting. (A) Real-time PCR using RNA isolated from C-siRNA cells and HMBOX1 siRNA cells showed that HMBOX1 mRNA levels were reduced by at least 70% in HMBOX1 siRNA3 cells, which was confirmed by Western blot analysis (B). (C) Data are shown as mean ± SEM from three independent experiments. **P*<0.05 and ***P*<0.01, compared to the C-siRNA group.
